# Scalable flibanserin nanocrystal-based novel sublingual platform for female hypoactive sexual desire disorder: engineering, optimization adopting the desirability function approach and *in vivo* pharmacokinetic study

**DOI:** 10.1080/10717544.2021.1938755

**Published:** 2021-06-26

**Authors:** Marianne J. Naguib, Amal I. A. Makhlouf

**Affiliations:** Faculty of Pharmacy, Department of Pharmaceutics and Industrial Pharmacy, Cairo University, Cairo, Egypt

**Keywords:** Flibanserin, nanocrystals, superdisintegrant, sublingual, desirability function, disintegration time, bioavailability

## Abstract

Flibanserin (FLB) was approved by FDA for the treatment of pre-menopausal female hypoactive sexual desire disorder (HSDD). FLB suffers from low oral bioavailability (33%) which might be due to hepatic first-pass metabolism in addition to its poor aqueous solubility. The sublingual route could be a promising alternative for FLB due to the avoidance of enterohepatic circulation. However, the drug needs to dissolve in the small volume of saliva in order to be absorbed through the sublingual mucosa. Therefore, FLB nanocrystals were prepared by sono-precipitation technique according to 2^3^ full factorial design. FLB-nanocrystals were formulated using two surfactants (PVP K30 and PL F127) in two different amounts (200 and 400 mg) and the volume of ethanol was either 3 or 5 mL. Nanocrystal formulation was optimized according to the desirability function to have a minimum particle size, zeta potential, polydispersity index, and maximum saturated solubility. The optimized formula had a particle size of 443.12 ± 14.91 nm and a saturated solubility of 23.27 ± 4.62 mg/L which is five times the saturated solubility of FLB. Nanocrystal dispersion of the optimized formula was solidified by freeze-drying and used to prepare rapidly disintegrating sublingual tablets containing Pharmaburst^®^ as superdisintegrant. Sublingual tablet formulation with the shortest disintegration time (36 s) was selected for the *in vivo* study. FLB nanocrystal-based sublingual tablets exhibited a two-fold increase in bioavailability with a faster onset of action compared to the commercially available oral formulation. These findings prove the potential application of FLB nanocrystal-based sublingual tablets in the treatment of HSDD.

## Introduction

Female hypoactive sexual desire disorder (HSDD) affects up to 30% of women in the USA (Clayton et al., 2018). It is described as persistent pre-menopausal reduced sexual desire that causes emotional distress and relationship difficulties and is not related to another mental condition or medication (American Psychiatric Association, 2013). Flibanserin (FLB) is the first FDA-approved medication for the treatment of HSDD (Joffe et al., 2016). The proposed mechanism of action of FLB is based on being an agonist of 5-HT_1A_ and antagonist of 5-HT_2A_ receptors. FLB increases the net norepinephrine concentration in the prefrontal cortex. The agonist effect at 5-HT_1A_ postsynaptic receptors increases the downstream concentration of dopamine and norepinephrine. Both neurotransmitters play a critical role in sexual excitation, sexual arousal, and boosting desire (English et al., 2017). However, the oral bioavailability of FLB does not exceed 33% (Joffe et al., 2016). This could be attributed to the extensive hepatic first-pass metabolism. Furthermore, FLB is a weak base characterized by variable solubility over the gastrointestinal tract (Clayton et al., 2020). It is highly soluble at pH 1.2 and insoluble at pH 6.8 (Rolf-Stefan & Julia, 2011). In addition, the oral absorption of FLB is reported to be greatly affected by food. Moderate fat meals increase the area under the curve of FLB by 1.5 folds which results in inter-and intra-subject variability in pharmacological response (Sprout Pharmaceuticals, 2015). Therefore, an alternative route of administration of the drug is of clinical significance.

Sublingual administration is a promising route for drugs whose absorption by the oral route is sub-optimal due to hepatic first-pass metabolism (Kotak & Devarajan, 2020; Leal-Júnior et al., 2020). Moreover, it usually provides rapid onset of action due to the high vasculature of the oral mucosa in addition to the ease of administration of sublingual tablets. However, the sublingual route is challenging for poorly water-soluble drugs because the drug needs to dissolve quickly in the limited volume of saliva to avoid being swallowed before absorption via the sublingual mucosa (Turunen et al., 2011). Rapidly disintegrating sublingual tablet can achieve this by incorporating hydrophilic polymers or superdisintegrants that allows the dosage form to disintegrate within a minute in the sublingual cavity upon contact with saliva (Song et al., 2018). However, after tablet disintegration, the drug needs to dissolve rapidly in saliva to be absorbed. Several strategies have been adopted to enhance the aqueous solubility of poorly soluble drugs such as solid dispersion and complexation (Turunen et al., 2011; Fong et al., 2015).

Nanocrystals dispersion is one of the most efficient and economically affordable approaches to enhance the solubility and consequently the bioavailability of drugs with poor water solubility (Rahim et al., 2017; Peltonen & Hirvonen, 2018). Reducing particle size would increase the surface area of drug particles which, according to Noyes–Whitney equation, increases the dissolution rate of drugs. Two strategies have been applied in the preparation of nanocrystals; top-bottom and bottom-top production. The top-down approach depends on particle size reduction using high-pressure homogenization or milling which is more energy-consuming, yet industrially applicable (Peltonen & Hirvonen, 2018). The bottom-top technique involves precipitation of drug particles from supersaturated solution using anti-solvent, solvent evaporation, or reduction of temperature. The bottom-top method consumes less energy and is more suitable for heat-sensitive substances (Zong et al., 2017).

In the present work, FLB nanocrystals were prepared using the antisolvent sono-precipitation technique. The effect of formulation factors on the characteristics of the prepared nanocrystals was studied applying 2^3^ factorial design. The optimized FLB nanocrystal formulation was solidified by freeze-drying and used to prepare fast disintegrating sublingual tablet containing PharmaBurst as superdisintegrant at the aim of improving the bioavailability of the drug and aiding in a faster onset of action. The prepared tablets were evaluated *in vitro* and the pharmacokinetic parameters were studied in rabbits compared to the commercially available FLB oral tablets.

## Materials

Flibanserin (FLB) was kindly supplied by Ramida Pharmaceuticals, Cairo, Egypt. Pluronic F127 (PL F127), sodium deoxycholate (SDC), Polyvinylpyrrolidone K30 (PVP) were purchased from Sigma-Aldrich Chemical Co. (St. Louis, USA). Pharmaburst^®^ (85% D-mannitol, <10% silicon dioxide, <10% sorbitol, 5% crospovidone) was a gift from Amoun Pharmaceuticals (Elobour, Egypt). Boric acid was purchased from El-Nasr Pharmaceutical Company (Cairo, Egypt). Other chemicals were of analytical grade and used as received.

### Methodology

#### Preparation of FLB nanocrystals (FLB-NC)

Flibanserin nanocrystals (FLB-NC) were prepared by the precipitation–ultrasonication method (Rahim et al., 2017). Firstly, FLB (100 mg) was dissolved in ethanol (3 or 5 mL) to form the solvent phase. Secondly, the solvent phase was injected into 20 mL of the antisolvent; precooled water (4^º^C) containing 100 mg SDC as a stabilizer and the surface-active agent (PL F127 or PVP K30) in different amounts in a 50 mL beaker. The inner diameter of the syringe used was 0.11 mm (insulin needle with a gauge of 29–31). Then, ultrasonication using probe sonicator at a power input of 100 W (VCX600, Sonics and Materials, Newtown, CT, USA) was applied for 10 min.

#### Experimental design

Three-factor two-level (2^3^) full factorial design was applied using Design-Expert^®^ 7 software (Stat-Ease, Inc., Minneapolis, MN) in order to optimize the formulation of FLB-NC and to study the effect of different formulation factors on the characteristics of the prepared nanocrystals. Based on preliminary trials, three independent variables, namely, type of surface-active agent (SAA), SAA amount, and the volume of ethanol were selected for the study. The particle size, polydispersity index, and zeta potential were the measured responses. Factors, their levels, and desirability constraints are listed in Table 1. Analysis of variance (ANOVA) was carried out to estimate the significance of the model and factors. Probability values (*p* <0.05) denoted significance. The detailed composition of the prepared formulations is listed in Table 2. All the formulae were prepared in triplicates and the results were expressed as mean ± SD.

**Table 1.** Independent variables and responses in 2^3^ full factorial design for FLB-NC preparation^a^.

Factors (independent variable)	Levels	
	−1	+1
X_1_: SAA type	PVP K30	PL F127
X_2_: SAA amount (mg)	200	400
X_3_: solvent volume (mL)	3	5
Responses (dependent variables)	Desirability constrains	
Y_1_: Particle size (nm)	Minimize	
Y_2_: Zeta potential (mV)	Minimize	
Y_3_: Polydispersity index	Minimize	
Y_4_: Saturated solubility (mg/L)	Maximize	

^a^All formulae contained 100 mg FLB and100 mg SDC (sodium deoxycholate). The antisolvent phase was 20 mL precooled distilled water.

FLB-NC: Flibanserin nanocrystals, SAA: surface active agent, PL F127: pluronic F127, PVP: polyvinyl pyrrolidone.

**Table 2.** Experimental runs, composition and responses for FLB-NC according to 2^3^ full factorial design.461.819

Formula code	Factors’ levels			Responses				Desirability
	X_1_	X_2_	X_3_	Y_1_	Y_2_	Y_3_	Y_4_	
	SAA type	SAA amount (mg)	Solvent volume (mL)	Particle size (nm)	Zeta potential (mV)	Polydispersity index	Saturated solubility mg/L	
FLB-NC1	PVP	200	3	493.32 ± 7.7	−21.05 ± 0.35	0.82 ± 0.21	21.61 ± 2.74	0.339
FLB-NC2	PVP	200	5	400.01 ± 69.30	−21.85 ± 1.48	0.74 ± 0.09	22.27 ± 1.60	0.655
FLB-NC3	PVP	400	3	640.45 ± 13.40	−22.75 ± 1.06	0.58 ± 0.21	21 93 ± 4.06	0.323
FLB-NC4	PVP	400	5	474.41 ± 8.83	−19.45 ± 4.45	0.70 ± 0.13	23.48 ± 4.50	0.643
FLB-NC5	PL F127	200	3	312.53 ± 4.60	−23.4 ± 0.42	0.86 ± 0.16	22.71 ± 1.16	0.435
FLB-NC6	PL F127	200	5	443.12 ± 14.91	−18.15 ± 2.62	0.42 ± 0.01	23.27 ± 4.62	0.762
FLB-NC7	PL F127	400	3	465.75 ± 35.60	−21.15 ± 1.77	0.68 ± 0.03	20.59 ± 1.83	0.448
FLB-NC8	PL F127	400	5	542.7 ± 30.41	−21.55 ± 2.19	0.51 ± 0.07	19.54 ± 2.33	0.656
FLB-NC6 (Predicted)	PL F127	200	5		−19.37	0.43		
Lyophilized FLB-NCT6	PL F127	200	5	462.45 ± 10.3	−17.35 ± 2.87	0.51±.07		

FLB-NC: Flibanserin nanocrystals; SAA: surface active agent; PVP: polyvinyl pyrrolidone; PL F127: pluronic F127.

### Characterization of FLB-NC

#### Particle size (PS), zeta potential (ZP) and polydispersity index (PDI) of FLB-NC

PS, ZP, and PDI were determined for FLB-NC formulae after appropriate dilution (1:20) using Zetasizer Nano ZS (Malvern Instrument Ltd., Worcestershire, UK) based on dynamic light scattering and electrophoretic mobility principles (Naguib et al., 2020). Measurements were performed thrice utilizing a glass cuvette at 25 °C with a count rate of 200–400 kilo counts per second (kcps) and the mean values ± standard deviations were recorded in Table 2.

#### Lyophilization of FLB-NC

FLB-NC were solidified by lyophilization (Ahmed et al., 2020). Nanocrystals dispersion was frozen at −20 °C overnight and subsequently freeze-dried at −45 °C for 24 h using the Novalyphe-NL500lyophilizer, Savant Instruments, Halprook, NY, USA. The lyophilized nanocrystals were kept in a desiccator over calcium chloride at room temperature for further investigations

#### Saturated solubility

The saturation solubility of pure FLB and the lyophilized FLB-NC formulae was determined in distilled water (Younes et al., 2021). Excess amounts of the tested samples were added to 5 mL distilled water in screw-capped glass vials. The vials were placed in a thermostatically controlled shaking water bath (GFL, Gesellschatt laboratories, Berlin, Germany) maintained at 100 strokes per min at 37 ± 0.5 °C. After 72 h, the supernatant was filtered through a 0.45μm millipore filter, and the amount of dissolved drug was analyzed spectrophotometrically at *λ*_max_ 255 nm using Shimadzu UV-1601 PC UV spectrophotometer, Japan after suitable dilution (Ahmed & Abdallah, 2020).

#### Optimization of FLB-NC

In order to select the optimum FLB-NC formula for further investigations, numerical optimization and desirability functions were employed utilizing Design-Expert^®^ software (El Assasy et al., 2019). FLB-NC formula with the highest desirability value (close to 1) was selected (Joseph Naguib et al., 2020).

### Characterization of lyophilized FLB-NC6

#### Particle size, polydispersity index, and zeta potential

The lyophilized FLB-NC6 was reconstituted with distilled water and investigated for its PS, ZP, and PDI. The measured responses were compared to the respective results obtained before lyophilization using Student’s *t*-test.

#### X-ray diffractometry (XRD)

X-ray diffractograms of pure FLB powder, a physical mixture of FLB, PL F127, and SDC in 1:2:1 ratio and the optimized nanocrystal formula (FLB-NC6) after lyophilization were obtained using an X-ray diffractometer (model XD-610, Shimadzu, Kyoto, Japan) at 45 kV and 40 mA. The 2*θ* angle range was between 100 and 600 with a scanning rate of 2°/min and a step size of 0.020.

#### Transmission electron microscopy (TEM)

FLB-NC6 was observed under a transmission electron microscope (JEM-1230, Jeol, Tokyo, Japan) operated at 80 kV. One drop was placed on a 400-mesh carbon-coated copper grid and stained with 2% Phosphotungistic acid leaving a thin liquid film that was air-dried before observation.

### Formulation of FLB nanocrystal-based sublingual tablets (FLB-NCT)

FLB-NCT were developed by direct compression technique. One hundred mg of the lyophilized FLB-NC6 powder containing 25 mg of FLB were blended with pharmaburst^®^, lactose, and boric acid in different ratios, and the total weight of the formulated tablets was set constant at 400 mg (Table 3). Powder blends were compressed using a single-punch machine (Royal Artist, Bombay, India) equipped with a flat bunch, 12 mm in diameter and an applied force of 4000 kg.

**Table 3.** Composition of FLB-NCT formulations^a^.

	FLB-NCT1	FLB-NCT2	FLB-NCT3	FLB-NCT4
Pharmaburst^®^	150	160	180	250
Lactose	150	90	100	
Boric acid		50	20	50
FLB-NC6	100	100	100	100
Disintegration time (s)	180 ± 9	154 ± 6	130 ± 18	36 ± 4

^a^FLB-NC6 100 mg contains 25 mg FLB. Weight of components expressed in mg.

Tablet weight was 400 mg.

FLB-NCT: Flibanserin nanocrystal-based sublingual tablets.

### Optimization of FLB-NCT

The optimum FLB-NCT formula for further investigations was selected based on the disintegration time according to USP specifications for sublingual tablets (<2 min) (Convention USP, 2012). The disintegration test was carried out for six tablets of each formula in distilled water at 37 ± 2 °C using the USP disintegration apparatus (Logan instruments incorporation, USA) without using the covering plastic disks. The tablets were kept in the baskets and the time taken for the tablet to disintegrate completely into smaller particles was noted (Convention USP, 2012).

### Evaluation of FLB-NCT4

#### Compressibility of FLB-NCT4 powder

The compressibility of FLB-NCT4 powder blend was evaluated to ensure that the powder is free-flowing with no problems during compression. Carr’s index and Hausner’s ratio are measures of powder compressibility. The lower the Carr’s index and Hausner’s ratio, the better is the powder flowability (Convention USP, 2012). Five grams of the powder was placed in a 25 mL graduated cylinder and the volume occupied was measured as *v_i_* (initial bulk volume) and used to calculate bulk density ($\rho_{b}).$ The cylindrical graduate was then tapped till a constant volume was obtained. The volume of the powder was then recorded as the final volume (*v_f_*) and used to calculate the tapped density ($\rho_{t}).$ Carr’s index and Hausner’s ratio were computed using the following equations (Salah et al., 2018):

(1) $$\rho_{b}=m/v_{i}\mathrm{.,}\rho_{t}=m/v_{t}.$$

(2) $$\mathrm{Car}{\mathrm{r'}}^{}\text{s index}=\frac{\rho_{t}-\rho_{b}}{\rho_{t}}\times 100$$

(3) $$\mathrm{Hausne}{\mathrm{r'}}^{}\text{s ratio}=\frac{\rho_{t}}{\rho_{b}}$$

Carr’s index less than 15, and Hausner’s ratio less than 1.8 indicate good compressibility according to the European Pharmacopeia (Council of Europe, 2017).

#### Characterization of FLB-NCT4

FLB-NCT4 was characterized for drug content, weight variation, thickness, hardness, and friability according to USP 37-NF 32 (Convention USP, 2012).

FLB content uniformity was tested by grinding 10 tablets individually and dissolving each tablet in ethanol. FLB content in each tablet was assayed spectrophotometrically at 255 nm and the average drug content was calculated. Weight variation was performed by accurately weighing 10 tablets individually, and the mean weight was calculated. The thickness and diameter of 10 tablets were measured using a micrometer (Vogel, Germany, DIN-863). The hardness was measured as the average breaking force of ten tablets measured by a hardness tester (TBH 325 series, Erweka GmbH, Heusenstamm, Germany). The mean hardness in kilograms was then determined.

A friabilator (Digital test apparatus, Model DFI-1; Veego, Bombay, India) was used to measure the friability of FLB-NCT4. Pre-weighed ten tablets were placed individually in the friabilator which rotated at 25 rpm for 4 min. Tablets were reweighed and percentage friability was calculated according to the following equation:

(4) $$\text{\% Friability}=\frac{\text{Loss in weight}}{\text{Initial weight}}\times 100.$$

No more than 1% loss from initial tablet weight to be accepted.

#### Wetting time and water absorption ratio of FLB-NCT4 (non-compendial)

The wetting time of the tablet was measured by placing 5 circular tissue papers (10 cm in diameter) in a Petri dish. Water (10 mL) containing methylene blue (0.1% w/v) was added to the Petri dish. A tablet was carefully placed on the surface of the tissue paper and the time required for the dye to reach the upper surface of the tablet was recorded as the wetting time. The tablet was weighed again and percentage weight gain was determined (Mehanna et al., 2020).

#### *In vitro* drug release

The release of FLB- NCT4 was performed using USP dissolution tester type II (rotating paddle, VK700 Dissolution Testing Station, Vankel Industries, Inc., NJ, USA) with a rotational speed of 50 rpm. *In vitro* release was carried out at 37 ± 1 °C in 250 mL Mcllvaine buffer (citric acid/phosphate buffer, pH 4). Aliquots were withdrawn in a predetermined time interval (5, 10, 15, 30, 60, 360 min.), filtered then assayed spectrophotometrically for FLB at *λ*_max_ 255 nm. The withdrawn samples were replaced with an equal volume of the release media. The cumulative drug released percent was plotted against time. The experiments were performed in triplicate for each sample.

### Statistical analysis of data

All measurements were conducted at least three times and expressed as mean ± standard deviation. Two-way analysis of variance (ANOVA) and the subsequent Bonferroni post hoc test or Student’s *t*-test was performed using SPSS version 23.0 (IBM Company, Chicago, USA) to detect significant differences between results. Differences were considered significant at *p* < 0.05.

### *In vivo* pharmacokinetic study

#### Animals

Ten male New Zealand albino rabbits were involved in the study. The rabbits (2–2.5 kg) were housed in separate cages kept in a light-controlled (alternate night and day cycles, 12 h each) air-conditioned chamber under controlled temperature and humidity (room temperature 25 ± 0.5 °C and ∼65% relative humidity). Feeding on standard laboratory diets (ad libitum and free access to water) was ensured. A medical checkup by a veterinarian was conducted before the beginning of the study to ensure the rabbits’ normal physical state and lack of clinically observable abnormalities.

#### Study design

Animal care and the experimental protocol were reviewed and approved by the Research Ethics Committee – Faculty of Pharmacy, Cairo University (REC-FOPCU) in Egypt (code PI 2765). A randomized two-period non-blind cross-over design was applied with a washout period of one week.

The rabbit dose was calculated by applying the following equation for dose translation based on body weight and surface area (Reagan‐Shaw et al., 2008):

(5) $$\text{Animal dose }(\mathrm{mg}/\mathrm{kg})=\text{Human dose }(\mathrm{mg}/\mathrm{kg})\times \frac{\mathrm{Human}K_{m}}{\mathrm{Animal}K_{m}}$$

Where *K_m_* values are 25 and 12 for human and rabbit, respectively. The rabbits were laid down in a horizontal position. The rabbit dose was found to be 4 mg/kg.

FLB-NCT4 tablets formed of 32 mg FLB-NC6 (8 mg FLB), 80 mg PharmaBurst^®^, and 16 mg boric acid with a total weight of 128 mg. The tablets were prepared following the same procedures mentioned before and compressed using the same tablet machine equipped with a 7 mm flat punch. The prepared tablets were characterized for disintegration time, drug content, weight uniformity, and friability and the results were quite matching with those for tablets containing 25 mg FLB (data not shown).

The rabbits were fasted overnight and on the study day, they were anesthetized using intramuscular injection of ketamine (25 mg/kg; Ketaminol, Intervet International B.V., Boxmeer, the Netherlands). The rabbits were then randomly allocated into two groups each of five rabbits.

The animals received an 8 mg single dose of FLB. The first group was administered FLB marketed product (Addyi^®^) orally, and the second group was administered FLB-NCT4 sublingually. For oral administration, Addyi^®^ tablets at a dose equivalent to 8 mg were given to the rabbits orally via catheterization, followed by rinsing the mouth with 4–5 mL normal saline solution (Turunen et al., 2011). For sublingual administration, the rabbit’s tongue was carefully lifted using a tweezer, and FLB-NCT4 tablet containing 8 mg FLB was placed under the tongue. The rabbit’s head was held in an upright position for 30 s post-administration to minimize swallowing (Sheu et al., 2016). Animals were fasted overnight and fasting continued for 4 h post-dose with free access to water.

Blood samples (2 mL) were collected into pre-heparinized tubes from the ear vein at 5, 10, 15, 30, 60, 360, and 1440 min after drug administration. Plasma was separated by centrifugation at **4**000 rpm at 5 °C for 20 min and stored in a freezer at −20 °C until being analyzed.

#### FLB quantitation in plasma

Plasma samples (300 µL) were mixed in the ratio of 1:3 with a 100 µL of flibaserin-d4 (500 ng/mL) as an internal standard and 4 mL of ethyl acetate. The mixtures were then vortexed, centrifuged and the organic layer was dried under vacuum. The residue was reconstituted with 300 µL of the mobile phase composed of acetonitrile and water +0.1% formic acid in the ratio of 8:2, respectively (He et al., 2019). The samples were eluted using HPLC apparatus (Shimadzu, Tokyo, Japan) equipped with Sunfire column (C18, 4.6 × 50 mm, 5 µm, Waters Corporation, Massachusetts, USA) at a flow rate of 0.98 mL/min. The method was validated with a linearity *R*^2^ = .9997, retention time of 0.373 min, and a lower limit of detection of 2 ng/mL.

#### Pharmacokinetic and statistical analysis

The *in vivo* results were processed using noncompartmental pharmacokinetic analysis generated by Kinetica^®^ software (Version 5, Thermo Fisher Scientific Inc., MA, USA) to determine the key pharmacokinetic parameters including FLB maximum concentration in plasma (*C*_max_), its corresponding time (*T*_max_), area under the curve from zero to the last sampling point (AUC_0–24_), and the area under the curve from zero to infinity (AUC_0–∞_). The relative bioavailability compared to the oral market product was calculated from the following equation:

(6) RB% =AUC0−∞(test)AUC0−∞(reference)×100.

The results were expressed as the mean of 10 rabbits ± SD. The pharmacokinetic parameters of FLB-NCT4 and the market product were compared using Student’s *t*-test at *p* < 0.05. Statistical analysis was performed using SPSS^®^ 23.0.

## Results and discussion

### Preparation of FLB-NC

Precipitation of drug nanocrystals utilizing an antisolvent is energy efficient and easily applied for a variety of drug candidates (Kassem et al., 2017). However, one of the major drawbacks of this method is the wide range of particle sizes of the formed nanocrystals, which can be overcome by applying ultrasonication during the precipitation of drug nanocrystals. Sono-precipitation provides control over the nucleation and crystallization process. Nanocrystals of FLB were prepared by dissolving the drug in ethanol followed by precipitation in an aqueous medium (Rahim et al., 2017). In order to assure precipitation of the drug in the nano range, a surface-active agent is incorporated in the antisolvent. Furthermore, sodium deoxycholate was added to impart surface negative charge on drug nanocrystals which induces electrical repulsion between particles and further stabilizes the system.

In order to study the effect of different formulation variables on the characteristics of the prepared FLB nanocrystals at the aim of optimizing the preparation conditions, 2^3^ full factorial design was applied. Three independent variables, namely, type of surfactant (PL F127 and PVP), amount of surfactant (200 and 400 mg), and the volume of ethanol (3 and 5 mL) were used to prepare FLB nanocrystals. Based on our preliminary experiments, sonication time was fixed to 10 min and the volume of aqueous phase was chosen to be 20 mL. All formulations contained 100 mg FLB dissolved in ethanol and 100 mg SDC dissolved in water.

### Characterization of FLB-NC

#### Particle size

The particle size of the prepared FLB nanocrystals ranged from 312.53 ± 4.60 nm to 640.45 ± 13.40 nm, which is comparable to a number of previous studies on nanocrystals preparation. For example, Dinda et al. prepared lacidipine nanocrystals with particle sizes ranging from 293.7 to 2530 nm applying high-pressure homogenization technique for 10 cycles, which is time and energy-consuming (Dinda & Panda, 2014). In another study, Kassem et al. prepared nanocrystal dispersion of the same drug by sono-precipitation technique with an average particle size range from 276.65 ± 47.45 to 1430.45 ± 44.9 nm (Kassem et al., 2017).

Analysis of variance (ANOVA) test for the effect of independent variables on the particle size of FLB-NC based on 2^3^ full factorial design revealed that the model was significant (F value equals 5.65). There is only 1.09% chance that the model F value could occur due to noise. The lack of fit was non-significant (*p* > 0.05) indicating the precision of statistical results. ANOVA test also demonstrated that the amount of surface-active agent has a significant effect on particle size (*p* < 0.05), whereas the type of surface-active agent and the volume of ethanol did not affect the particle size in a statistically significant manner (*p* >0 .05).

**Figure 1.**
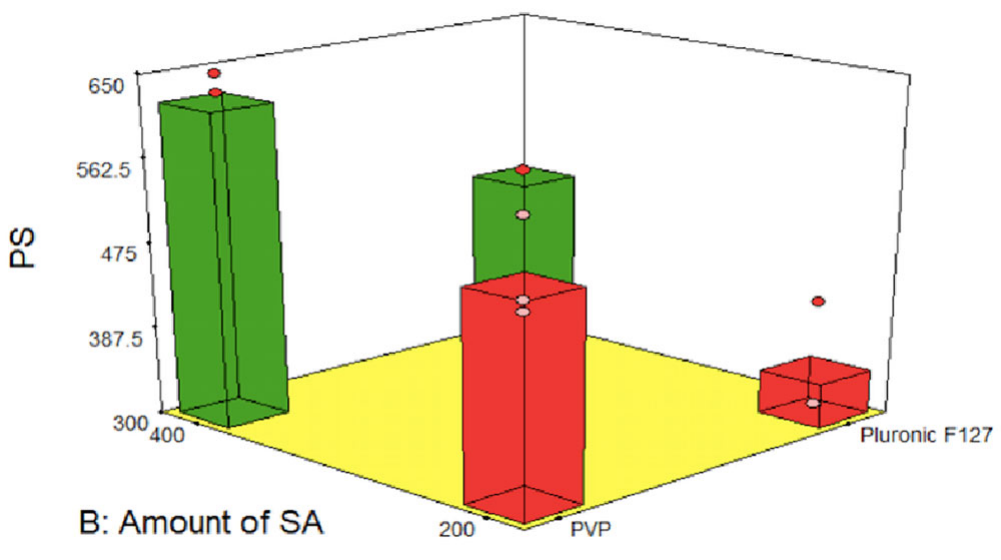
Effect of surface active agent type and surface active agent amount on the particle size of FLB-NC. FLB-NC: Flibanserin nanocrystals.

As shown in Figure 1, smaller FLB nanocrystals size was imparted by lower surfactant concentration. However, optimizing surface-active agent concentration is crucial in producing nanocrystals with the smallest possible particle size. Surfactant molecules at the appropriate concentration are adsorbed on particles’ surface forming a mechanical barrier against crystal growth leading to the formation of small nanocrystals (Hao et al., 2015). On the other hand, excess surfactant could increase the thickness of the adsorbed protective layer leading to a larger particle size measured on the zeta sizer. Furthermore, increasing the surfactant level above the critical micelle concentration leads to the assembly of surfactant molecules to form micelles rather than being adsorbed on the surface of particles. In addition, micelles formation can solubilize the drug and by time increase particle size by Ostwald ripening (Sinha et al., 2013).

#### Polydispersity index

Polydispersity index is a measure of the homogeneity of particle size within the dispersed system. The smaller the dispersity index, the more uniform the system (Salah et al., 2018). The PDI of the prepared FLB-nanocrystals ranged from 0.42 ± 0.01 to 0.86 ± 0.16 (Table 2). The results came in harmony with previous research involving the preparation of nanocrystals by sono-precipitation method and got similar PDI values (Kassem et al., 2017). Similarly, Xia et al., tried to formulate nitrendipine nanocrystals using the sonication method and reported that increasing drug concentration and greater supersaturation resulted in higher crystal growth and agglomeration rate, leading to larger initial crystals with an increase in the PDI value (Xia et al., 2010). However, the ANOVA test revealed that there was a non-significant difference in PDI values between formulations (*p* > 0.05).

#### Zeta potential

Zeta potential measures the surface charge on the particles which increases the stability of the dispersion by imparting electrical repulsion between nanocrystals. Sodium deoxycholate was incorporated in FLB nanocrystals preparation, which was responsible for the negative zeta potential values of the resultant particles. Zeta potential for all the prepared formulations was approximately −20, which is enough to impart stable nanocrystal dispersion (Honary & Zahir, 2013). There was a non-significant difference in zeta potential between formulations (*p* > 0.05) which can be explained by the fixed amount of SDC in all formulations (100 mg).

#### Saturated solubility

Saturation solubility of the prepared FLB-NC is presented in Table 2. The saturated solubility of FLB-NC ranged from 19.54 ± 2.33 mg/L to 23.48 ± 4.5 mg/L, which is about five times the aqueous solubility of pure FLB (4.32 ± 0.56 mg/L). Decreasing the particle size to the nano-range increased the surface area in contact with the solvent and consequently increased drug solubility. Moreover, the enhanced solubility of FLB-NC could be attributed to the decrease in crystallinity of the drug as revealed in the diffractogram of the FLB-NC-6 as will be seen later. However, the difference in the saturation solubility between FLB-NC formulations was non-significant (*p* > 0.05).

#### Optimization of FLB-NC

The desirability function is an effective approach in the optimization of pharmaceutical preparations in which several variables affect the characteristics of the final formulation (Nour et al., 2015; Salah et al., 2018). The desirability constraints in FLB-NC were minimum particle size, minimum polydispersity index, and maximum zeta potential. According to the desirability values (Table 2), FLB-NC6, with a desirability value of 0.762 was the optimum formulation. The predicted values for the dependent variables, as calculated by Design Expert^®^ software, came in good agreement with the observed results as shown in (Table 2). Therefore, nanocrystal formulation FLB-NC6 composed of 200 mg Pluronic F127, 100 mg sodium deoxycholate, 100 mg FLB and prepared using 5 mL of ethanol was chosen for further investigations.

### Characterization of FLB-NC6

#### Particle size, polydispersity index, and zeta potential

Lyophilization is an effective technique to increase the stability of liquid dosage forms by sublimation of the aqueous phase under reduced pressure. Furthermore, solidification of a liquid dosage form enables filling it inside hard gelatin capsules or compression with other excipients in the form of tablets (El Assasy et al., 2019). At the aim of preparing nanocrystal-based sublingual FLB tablets, the optimized formula (FLB-NC-6) was lyophilized and the characteristics of the lyophilized nanocrystals after reconstitution were evaluated. There was a non-significant difference (*p* < 0.05) in PS, ZP, and PDI of FLB-NC6 before and after lyophilization (Table 2) as determined by the Student’s *t-*test.

#### X-ray diffractometry (XRD)

**Figure 2.**
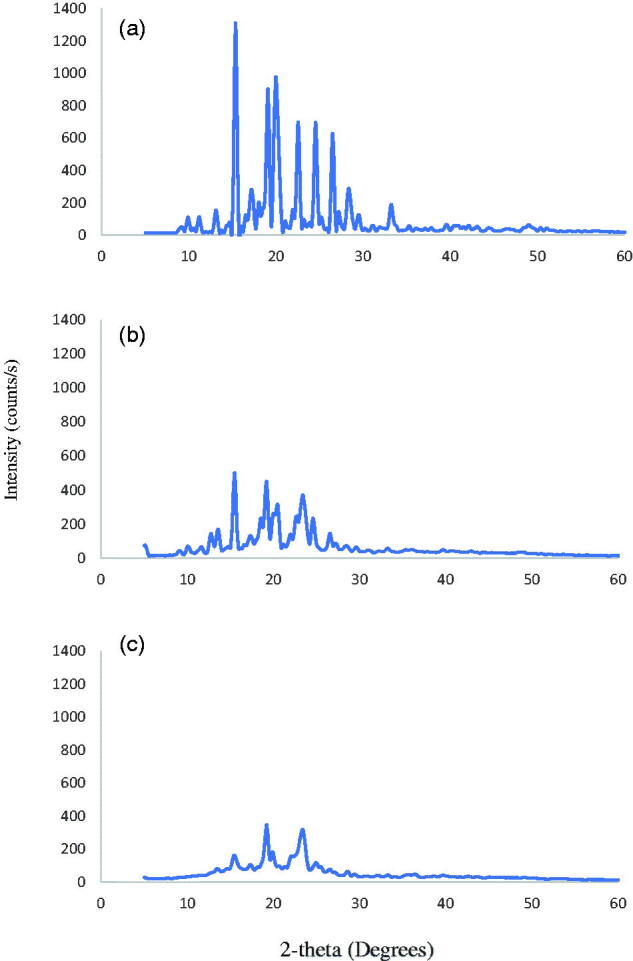
X-ray diffractograms of (a) pure FLB; (b) FLB:Pluronic F127:sodium deoxycholate 1:2:1 physical mixture, and (c) FLB-NC6.

The diffractograms of pure FLB, the physical mixture of FLB, PL F127, and SDC in 1:2:1 ratio and the optimized nanocrystal formula FLB-NC6 after lyophilization are presented in Figure 2. The crystalline nature of pure FLB powder was demonstrated by the intensity of the peaks in the diffractogram of the drug. However, the intensity of the peaks was diminished in the diffractogram of the physical mixture due to the dilution of the dug by the other components. On the other hand, a further decrease in peak intensity was observed in the thermogram of FLB nanocrystals formulation indicating phase transition to less crystalline form during the precipitation of the nanocrystals. The formation of a less crystalline form of the drug might be attributed to the fast nucleation during nanocrystals precipitation which did not give enough time to drug particles to form perfect crystals (Hao et al., 2015). The decrease in drug crystallinity might contribute to the increase in saturation solubility and dissolution rate of the FLB nanocrystals.

#### Transmission electron microscopy (TEM)

**Figure 3.**
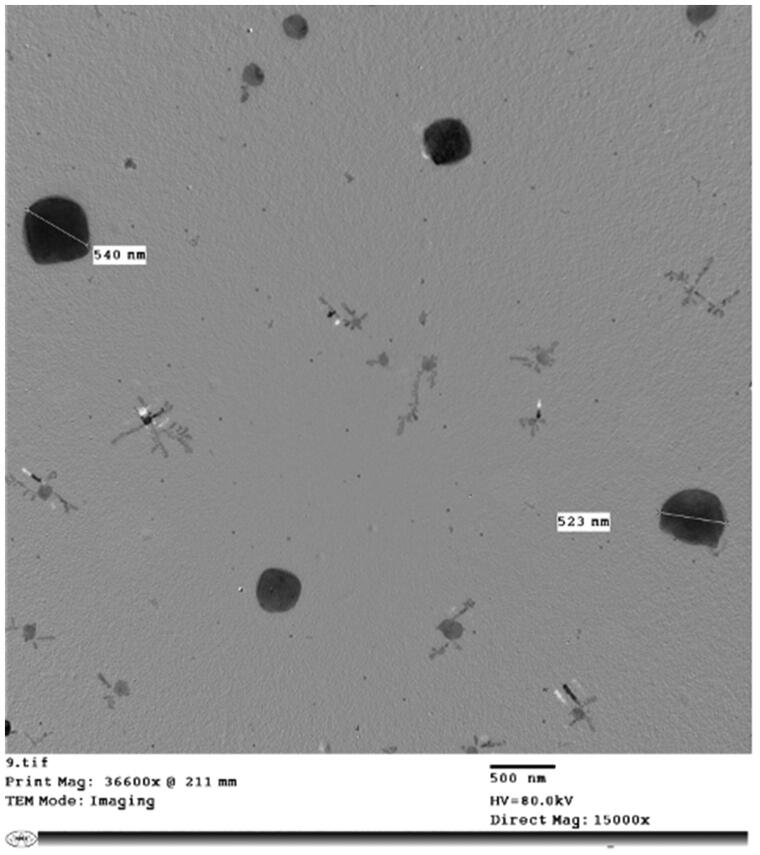
Transmission electron microscopic image of FLB-NC6.

Figure 3 visualizes the FLB-NC6 under a transmission electron microscope. It is clear that the particles are spherical in shape with a particle size average of 500 nm, which came in good agreement with Zetasizer measurements. A similar observation was reported by Kassem et al. who observed the spherical shape of lacidipine nanocrystals (Kassem et al., 2017).

#### Preparation of FLB nanocrystal-based sublingual tablets (FLB-NCT)

Various techniques have been applied in the preparation of orodispersible tablets. Direct compression is one of the most widely used methods due to its ease of preparation and industrial applicability. In addition, it does not require the addition of water or organic solvent (Rawas-Qalaji et al., 2006). However, formulating sublingual tablets of a poorly water-soluble drug is quite challenging as the drug needs to dissolve rapidly in the small volume of saliva. This necessitates formulating the drug in a form that increases drug solubility and the incorporation of superdisintegrant and water-soluble excipients in tablet formulation. In our study, FLB nanocrystals with higher saturated solubility than pure drugs were prepared and optimized. The lyophilized nanocrystal formula FLB-NC6 was used in the preparation of FLB sublingual tablets. In addition, Pharmaburst^®^ (85% D-mannitol, <10% silicon dioxide, <10% sorbitol, 5% crospovidone) (Dziemidowicz et al., 2018) was used as superdisintegrant. Pharmaburst provides good patient acceptability and produces tablets with mechanical strength that withstand transportation, storage, and removal from the blister, and at the same time rapidly disintegrates in the mouth after administration (Yousef et al., 2005; Tayel et al., 2017).

Since FLB is a weakly basic drug, the incorporation of acidic excipient might provide an acidic microenvironment surrounding the tablet which increases drug solubility (Younes et al., 2021). Therefore, boric acid was chosen as a lubricant in the prepared FLB sublingual tablets. In addition to being used as a lubricant (Li & Wu, 2014), it might improve the aqueous solubility and dissolution of the drug (Adachi et al., 2015; Rakkaew et al., 2018).

#### Characterization of FLB-NCT

Disintegration time is crucial in the development of sublingual tablets. According to USP, the disintegration time of sublingual tablets should not exceed 2 min. Table 3 demonstrates the disintegration time of the prepared FLB-NCT. The disintegration time ranged from 36 ± 4 to 180 ± 9 s, and the formulae came in the following order FLB-NCT1 > FLB-NCT2 > FLB-NCT3 > FLB-NCT4. The disintegration time of the prepared tablets increased with the decrease in Pharmaburst^®^ content in the formula. This is most probably attributed to the presence of crospovidone as a component of Pharmaburst^®^. Previous research reported a disintegration time of less than 40 s for sublingual tablets containing crospovidone (Khinchi et al., 2011). Accordingly, FLB-NCT4 was chosen for further investigations.

Wetting time is an important test for sublingual orodispersible tablets as it simulates the condition under the tongue with small saliva volume ranging from 1 to 5 mL (Rawas-Qalaji et al., 2006). It also gives an indication about the porosity of the tablets, and it was found to be correlated with the disintegration time (Mehanna et al., 2020). The wetting time of FLB-NCT4 was found to be 36 s, which is quite matching with the disintegration time result.

Weight variation, content uniformity, and friability of FLB-NCT came within the acceptable limits as per USP specifications (Convention USP, 2012). The weight of 10 tablets was 400 mg ± 10, and the FLB content was 25 mg ± 5%. The thickness and diameter of ten tablets were 3 and 12 mm, respectively, with a non-significant difference between tablets (*p* > 0.05).

Friability is an evaluation of the effect of friction which may occur during handling of the tablet and may result in tablet chip or breakdown, whereas hardness is the force required to crush the tablet. Friability was found to be less than 1% (0.07%), and hardness was found to be 2.5 kg which indicated that FLB-NCT4 has good mechanical resistance. The relatively low hardness is important in sublingual tablets to rapidly disintegrate in the small volume of saliva in the sublingual cavity (Tayel et al., 2017).

#### Compressibility of FLB-NCT4

The measurement of compressibility index (Carr’s index) and Hausner’s ratio is a fast and easy method to predict the flowability and consequently the compressibility of powders (Convention USP, 2012). They depend on bulk and tapped density which is related to the cohesiveness and moisture content of the powder (Council of Europe, 2017). The values of Carr’s index and Hausner’s ratio are 14% and 1.16, respectively, indicating good flow property as per the European Pharmacopeia 6.0 (Table 4) (Council of Europe, 2017).

**Table 4.** Compressibility parameters of FLB-NCT4.

Parameter	Value
Bulk density	0.136 g/mL
Tapped density	0.157.89 g/mL
Carr’s index	14%
Hausner’s ratio	1.16

FLB-NCT: Flibanserin nanocrystal-based sublingual tablet.

#### *In vitro* dissolution study

**Figure 4.**
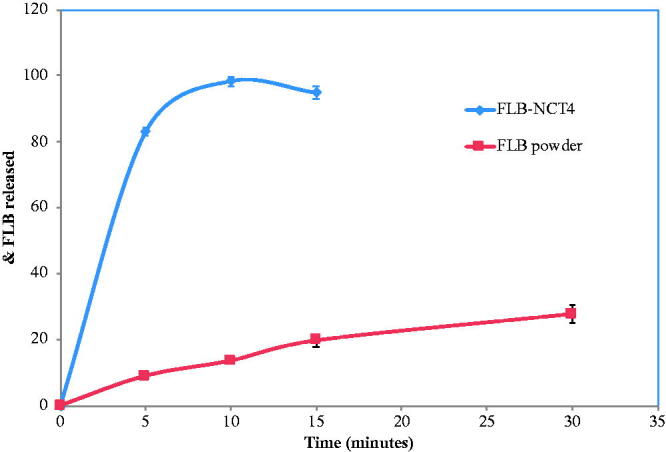
*In vitro* dissolution profiles of FLB-NCT4 and FLB powder in McIlvaine buffer pH 4. FLB-NCT4: Flibanserin nanocrystal-based sublingual tablet.

The dissolution test of FLB-NCT4 was carried out in 250 mL McIlvaine buffer of pH 4 to ensure sink condition according to the US Food and Drug Administration guidelines for the dissolution testing of FLB (USFDA, 2021). It was found that approximately 80% of the drug was released within 5 min and that complete dissolution was within 10 min compared to less than 40% drug release within 1 h for FLB powder. (Figure 4). This came in accordance with previous literature which specified that the amount of drug dissolved from sublingual tablets should exceed 80% in 15 min (Klancke, 2003; Bayrak et al., 2011). A number of factors contributed to the fast dissolution of rate of FLB-NCT4. First of all, the nano-sized particles increased the saturated solubility of the drug almost 6 times from 4 to 23 mg/L as shown above. The decrease in particle size is accompanied by an increase of the total surface area exposed to the dissolution medium which consequently increased the dissolution rate of the drug according to Noyes–Whitney equation (Lippincott Williams & Wilkins, 2011):

(7) $$\frac{\mathrm{dc}}{\mathrm{dt}}=A\times D\frac{\left(Cs-Cb\right)}{h}$$

where dc/dt is the dissolution rate, *A* is the surface area, *D* is the diffusion coefficient of the drug, *C_s_* is the saturation solubility, *C_b_* is the bulk concentration, and *h* is the diffusional path. Particle size reduction increases concentration gradient and decreases the diffusion distance which consequently increases the dissolution rate. A similar observation was recorded by Kassem et al. who reported complete dissolution of lacidipine nanocrystals within 120 min compared to the raw drug which showed almost no dissolution within the same time frame (Kassem et al., 2017). In addition, the incorporation of boric acid in the formula decreased the pH in the microenvironment surrounding the tablet which provided favorable conditions for drug dissolution and increased the dissolution rate. These results coincided with those reported by Younes et al. who found that the incorporation of organic acid as a pH modifier imparted pH-independent release of the weakly basic amisulpride (Younes et al., 2021). Furthermore, the decrease in drug crystallinity, which was proven by X-ray diffractometry, fastened the dissolution rate. Another key factor in drug dissolution is the rapid disintegration of the tablets (36 s) due to the presence of Pharmaburst^®^ (60% of tablet weight) which acted as superdisintegrant. Tablet disintegration results in the dispersion of the tablet in the form of a suspension in the dissolution medium which fastened drug release. These results came in accordance with Moqbel et al. who found that pharmaburst^®^ was the most efficient superdisintegrant among the tested ones, and it produced 100% chlorzoxazone release within 15 min (solubility of chlorzoxazone in water 1000 mg/L) (Moqbel et al., 2016).

#### *In vivo* pharmacokinetic study

Several animal models, such as pigs, sheep, monkeys, rabbits, and dogs, have been adopted to test the pharmacokinetics of sublingual dosage forms. These animals have non-keratinized sublingual epithelium that resembles human sublingual mucosa (Turunen et al., 2011). In the present study, rabbits were employed as an animal model due to their convenient size, affordability, and ease of handling.

**Figure 5.**
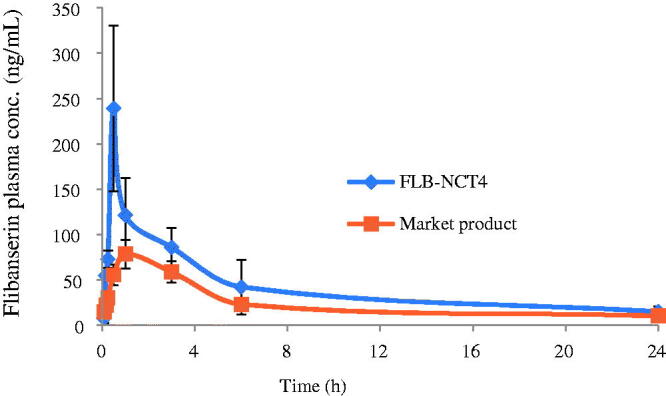
Plasma-concentration time profile of FLB in rabbits after sublingual administration of FLB-NCT4 compared to the oral market product (Mean ± SD, *n* = 10). FLB-NCT: Flibanserin nanocrystal-based sublingual tablets.

Furthermore, rabbits have been used to study the sublingual absorption of a number of weakly basic drugs such as propranolol, verapamil, captopril, and midazolam (Odou et al., 1999; Dali et al., 2006). Rabbit’s dose was calculated according to Reagan-Shaw et al. based on both body weight and body surface area and found to be approximately 4 mg/kg (Reagan‐Shaw et al., 2008). Figure 5 shows the mean plasma concentration-time profile of FLB (4 mg/kg) after sublingual administration of FLB-NCT4 compared to the oral market product. The corresponding pharmacokinetic parameters including *C*_max_. *T*_max_, AUC_0–24_, AUC_0–∞_ and *T*_1/2_ are listed in Table 5.

**Table 5.** Pharmacokinetic parameters of FLB in rabbits after sublingual administration of FLB-NCT4 compared to the oral market product (Mean ± SD, *n* = 10).

Pharmacokinetic parameter	FLB-NCT4	Market product
*C*_max_ (ng/mL)	270.559 ± 80.56*	79.437 ± 3.81
*T*_max_ (h)	0.49 ± 0.11*	1.29 ± 0.42
AUC_0–24_ (ng/mL h)	1242.076 ± 206.26*	560.530 ± 182.03
AUC_0–∞_ (ng/mL h)	1437.73 ± 343.90*	675.877 ± 166.04
*K* (h^−1^)	0.0614 ± 0.001	0.0596 ± 0.001
*T*_1/2_ (h)	11.28 ± 1.98	11.61 ± 1.13
Relative bioavailability	2.12	

*Significant difference (*p* < 0.05).

FLB-NCT: Flibanserin nanocrystal-based sublingual tablets.

Two-Way ANOVA test revealed that neither the period nor the rabbits had a significant effect on the pharmacokinetic parameters (*p* >0 .05). Maximum plasma concentration (*C*_max_) of FLB-NCT4 (270.559 ± 80.56 ng/mL) was three times greater than that of the oral market product (79.437 ± 3.81 ng/mL) which was determined to be statistically significant (*p* < 0.05). The time to reach maximum plasma concentration (*T*_max_) of FLB-NCT4 was 0.49 ± 0.11 h compared to 1.29 ± 0.42 h for oral FLB (*p* < 0.05). There was a non-significant difference in half-life (*T*_1/2_) and elimination rate constant (*K*) between oral and sublingual routes (*p* > 0.05). However, there was a two-fold increase in AUC_0–24_ and AUC_0–∞_ for nanocrystal-based sublingual tablets compared to conventional oral tablets (statistically significant at *p* <0 .05). Therefore, the relative bioavailability of FLB-NCT4 was approximately 200% compared to the oral market product.

A primary reason for the enhanced bioavailability of the sublingual tablets compared to the oral ones is the avoidance of first-pass metabolism. FLB is reported to suffer from extensive hepatic first-pass effect resulted in low oral bioavailability (30%). In addition, sublingual mucosa is five times thinner than buccal mucosa, and this makes it the most permeable region of the oral cavity (Goswami et al., 2008). However, in the sublingual cavity, the drug is required to dissolve in a small volume of saliva (∼1mL) in order to be available for absorption. This is challenging for the poorly water-soluble FLB (aqueous solubility of FLB is 4.312 mg/L). Reducing the particle size of FLB to the nano-range was pivotal to increase the aqueous solubility of the drug and therefore enhance its sublingual absorption. In addition, the incorporation of boric acid in the formula decreased the microenvironmental pH surrounding the tablet which enhanced the dissolution of the weakly basic FLB. Furthermore, the enhanced bioavailability of FLB from the prepared sublingual nanocrystal-based tablets can give the chance to decrease the daily dose of FLB. The simple preparation procedure and the economically efficient excipients make FLB nanocrystal-based tablets a more affordable alternative to the commercially available oral tablets for the treatment of female hypoactive sexual desire disorder.

## Conclusion

The present work aimed at increasing the bioavailability of FLB (33% bioavailable). Sublingual administration is a favorable option for FLB as it is subjected to intensive first-pass metabolism. However, due to its poor aqueous solubility, the sublingual route was quite challenging for FLB. FLB-Nanocrystal dispersion was prepared and optimized applying the desirability function approach and the saturated solubility was improved by five folds. The nanocrystal dispersion was solidified by freeze-drying and FLB sublingual tablets were prepared by direct compression. The disintegration time of the tablets was 36 s. The *in vivo* pharmacokinetic study revealed a two-fold increase in the bioavailability of FLB from the nanocrystal-based sublingual tablets. Based on our findings, FLB nanocrystal-based sublingual tablets are a promising platform that would increase the bioavailability of the drug, provide faster onset of action, and avoid inter and intra-subject variability accompanied by the oral administration of the drug. Furthermore, the preparation technique is simple and the excipients used are economically efficient, which provides an additional advantage for industrial production.
